# Cytotoxicity of Self-Etch Versus Etch-and-Rinse Dentin Adhesives After 48 h: An In Vitro Study

**DOI:** 10.3390/dj14050312

**Published:** 2026-05-19

**Authors:** Kamelia Parkhoo, Lea Aylin Schmitz, Luisa Fröb, Nicole Grüßner, Georgios E. Romanos, Eva Herrmann, Susanne Gerhardt-Szép

**Affiliations:** 1 Department of Operative Dentistry, Carolinum, Goethe University Frankfurt, Theodor-Stern-Kai 7, 60590 Frankfurt am Main, Germany; le.schmitz@em.uni-frankfurt.de (L.A.S.); luisa.froeb@gmx.net (L.F.); gruessner@med.uni-frankfurt.de (N.G.); 2 Department of Oral Surgery and Implant Dentistry and Oral Medicine, Carolinum, Goethe University Frankfurt, Theodor-Stern-Kai 7, 60590 Frankfurt am Main, Germany; 3 Institute of Biostatistics and Mathematical Modelling, Goethe University, Theodor-Stern-Kai 7, 60590 Frankfurt am Main, Germany; herrmann@med.uni-frankfurt.de

**Keywords:** cells, materials testing, fibroblasts, in vitro techniques, adhesives

## Abstract

**Objectives**: Six dentin adhesives were tested in vitro regarding their cytotoxicity toward human fibroblasts. AdheSE, Clearfil SE Bond, Hybrid Bond, One-up Bond F Plus, Optibond Solo Plus, and Syntac were tested using a cell culture model. The several components of dentin adhesives, like the primer and bonding, were analyzed as single and additive applied components as specified by the manufacturer for application in vivo. **Methods**: Seventy-five Petri dishes were produced per adhesive and control group, and all 525 Petri dishes were evaluated using multiparametric strategies, i.e., using multiple methods to strengthen the reliability of the results. The multiparametric strategies consisted of automated cell counting for viability, microscopic morphological assessment and lastly of reactivity grading according to ISO 10993-5. These assessments were performed after our initial investigation, and the observation period was extended from 24 h to 48 h. **Results**: AdheSE, Clearfil SE Bond, One-up Bond F Plus, and Optibond Solo Plus showed statistically significant reductions in viable cells relative to the cell control. All dentin adhesives except Clearfil SE Bond showed a statistically significant difference regarding the reactivity index in the application comparison. **Conclusions**: The test materials showed a moderate degree of cytotoxicity, with no statistically significant difference between the tested self-etch and etch-and-rinse dentin adhesives. However, the results show statistically significant differences between the adhesives when applied sequentially and once. Further research addressing mechanisms of cytotoxicity is needed for advancement in this field.

## 1. Introduction

Resin-based composites (RBCs) have replaced amalgam as the gold standard of dental restorative materials [1,2,3,4]. RBCs are advantageous because of their adhesion to the tooth, which allows for minimally invasive procedures and excellent aesthetics through tooth-colored restorations. Dentin adhesives function as intermediary connections between dentin and RBCs. They are a solvated monomeric blend of lipophilic and hydrophilic components [5]. Through research, several dental adhesive approaches have been established, such as etch-and-rinse and self-etch systems. Self-etch dentin adhesives reduce the number of application steps by leaving out the separate process of conditioning the tooth structure and thus saving on treatment time [6,7,8,9]. However, the monomers contained in adhesive systems have also been the subject of research regarding possible cytotoxic and genotoxic effects [3,10,11,12].

It is important to investigate the cytotoxicity of dentin adhesives according to ISO standards, as these adhesives come into contact with intraoral tissues such as gingival or pulp cells during intraoral application [10,13]. Studies have already demonstrated that dentin adhesives can cause cell damage [10,14,15,16].

In our first study, the eluates of six different dentin adhesives were tested for their cytotoxicity in vitro toward cell cultures with primary fibroblasts after an observation period of 24 h [17]. This screening study showed an initial ranking of cytotoxicity based on qualitative assessments of dentin adhesives [17,18,19]. In order to lean more towards in vivo situations where residues of adhesives may engage in prolonged cell contact, a longer period should be tested, according to ISO 10993-5 [18]. Furthermore, determining whether there are differences in the types of adhesives over an extended period is of clinical interest. Therefore, this study functions as an extension of our first study, in which the same dentin adhesives were investigated, but now for a period of 48 h in order to observe cellular reactions beyond the initial reaction [20].

The critical feature of our study remains the multiparametric evaluation of cytotoxicity [17,21]. In order to more accurately determine if in vitro cytotoxicity is unsuitable for clinical in vivo situations, authorities recommend performing several biocompatibility tests to obtain a more precise assessment of the potential biological risk associated with clinical use of the materials [22]. No one testing method offers universally reliable results; thus, multiple strategies combining at least two different endpoints capture different aspects of cytotoxicity [21]. While quantitative evaluation offers viability data, qualitative analysis captures structural changes, which are not reflected in cell counts. Further, the reactivity index offers facilitated interpretation within a framework established by ISO 10993-5. Therefore, it is precisely the combination of qualitative and quantitative assessments that is crucial for obtaining more detailed conclusions. As in our first study, comparison with existing studies is difficult because our methods and parameters cannot be found in other studies [11,16,23,24,25,26,27,28,29,30,31,32,33,34,35,36,37,38,39,40]. In several studies, cytotoxicity was detected only after 24 h [16,27,28,30,34,36,37,38,39]. These studies show the short-term effect of the cytotoxicity of dentin adhesives. However, only a few studies provide results obtained after the combination of different observation periods, as recommended according to ISO 10993-5 [18,20,24,26,32,35,40,41,42].

In this study, we compared and evaluated the cytotoxicity of self-etch and etch-and-rinse dentin adhesives after 48 h, allowing comparison with the first observation period of 24 h.

The hypotheses for our screening study are as follows:

- The self-etch adhesives will show no differences in cytotoxicity relative to the etch-and-rinse adhesives in the multiparametric evaluation.
- The sequentially applied substances will have different cytotoxic effects relative to those applied once in the multiparametric evaluation.

## 2. Materials and Methods

### 2.1. Materials and Cells

Six different dentin adhesives were evaluated: AdheSE (Ivoclar Vivadent, Schaan, Liechtenstein), Clearfil SE Bond (Kuraray, Okayama, Japan), Hybrid Bond (Sun medical, Moriyama, Japan), One-up Bond F Plus (Tokuyama, Tokyo, Japan), Optibond Solo Plus (Kerr, West Collins, Orange, CA, USA, USA), and Syntac (Ivoclar Vivadent, Schaan, Liechtenstein). Explants of normal human gingival tissue (Ethical approval code: 275/07; approval date: 12 October 2007) acquired through surgical periodontal operation were obtained from the Department of Oral Surgery and Implant Dentistry (Carolinum, Goethe University Frankfurt). The obtained material was stored overnight in Hanks balanced salt solution (Gibco-Life Technologies Ltd., Paisley, Scotland) in a refrigerator at 4 °C to cleanse the explants of blood and granulation tissue. They were supplemented with five milliliters of bicarbonate and an antibiotic additive and cooled to 4 °C (Gibco-Life Technologies Ltd., Paisley, Scotland) in order to achieve conditions that were as antibacterial as possible. To prepare the human tissue for the tests, it was cut with a sharp scalpel (No. 15, Aesculap, Tuttlingen, Germany) into small, uniform pieces measuring 1 mm^3^. Afterwards, the explants were transferred to surface-treated 50 cm^3^ polystyrene culture bottles and 50 mm diameter polystyrene Petri dishes (Falcon, Becton and Dickinson, Heidelberg, Germany). The test tissue was dried for one to two minutes at room temperature. Afterwards, five milliliters of the culture medium (BM Eagle–Basal Medium) and 10% calf serum (both materials: Gibco, Paisley, Scotland, UK) were added to each Petri dish. Because oral human tissue cannot be obtained under sterile conditions, the germ content must be reduced as much as possible with antibiotics. Penicillin was applied to every culture medium. The Petri dishes were incubated in a gas incubator (Nr, Heraeus, Hanau, Germany) at 37 °C in a 4.5% CO_2_ atmosphere with a humidity of 95%. The culture medium was initially renewed every five to seven days and then after two to three days. The first proliferation of epithelial and fibroblast cells around the explants occurred after 18 to 24 days, and after two to three days, a cell monolayer could be observed. For this investigation, a pure fibroblast culture was obtained via trypsinization. The dentin adhesives were applied in the center of sterile bases of glass slides under antibacterial conditions. The glass slides with the single adhesive part remained uncured. Meanwhile, successively applied adhesive parts were cured with the aid of an Elipar II curing light (ESPE, Seefeld, Germany). The curing times were set according to the respective instructions for use as recommended by the manufacturer. These prepared glass slides were weighed and placed in the center of each Petri dish. Afterwards, the culture medium was applied to Petri dishes to achieve a concentration of 0.2 milligrams of adhesive per one milliliter of medium. The extraction concentration was the same as that used in earlier research and is not in accordance with ISO 10993-5 [8]. Five milliliters of this eluate was applied to fibroblasts in Petri dishes that had been left to stand for 24 h. They were between the 8th and 18th passage. Then, these Petri dishes were incubated at 37 °C in a 4.5% CO_2_ atmosphere for 48 h. Finally, the cell cultures were fixed with 98% pure ethanol and stained with Pappenheim’s panoptic stain. This method was used in previous research [8,43]. Each Petri dish is treated as independent as no adhesive component is linked to the others. The test materials and methods, as well as the study design, are identical to those from our first study [17].

### 2.2. The Multiparametric Strategies Tested

#### 2.2.1. Quantitative Evaluation

A total of 105 Petri dishes, 15 per adhesive group and 15 for the control group, representing each adhesive and the control, were used for the quantitative assessment. Each sample was evaluated as being “viable”, “dead,” or “debris” after 48 h using a cell counter (Cell-Counter CASY DT, OLS GmbH and Co KG, Bremen, Germany). The values were adjusted after establishing the initial settings for fibroblasts: viable: 12.8–100 µm; dead: 7.6–12.8 µm; debris: 3.3–7.7 µm. The values for “viable”, “dead,” and “debris” fibroblasts are based on manufacturer recommendations.

#### 2.2.2. Qualitative Evaluation

In addition, a total of 420 Petri dishes, with 60 per group, were used for the qualitative analysis. They were examined at 100–250-fold magnification under a contrasting phase microscope (Leica, Bensheim, Germany) to identify physiological and pathological cellular changes. Predefined evaluation criteria included the general morphology, reactions, and growth of the fibroblasts were evaluated, along with any vacuolization, detachment, and cell lysis that may have occurred. To document these cellular changes, 420 photos of the cell cultures were taken. Two trained observers assessed the fibroblasts.

#### 2.2.3. Reactivity Index

Based on the qualitative evaluation, the reactivity index was assessed according to ISO 10993-5 (Table 1) [18].

### 2.3. Statistical Analysis

The data were evaluated using Kruskal–Wallis multiple Conover–Iman comparison with Bonferroni–Holm corrections (BiAS. 11. 10, Epsilon, Frankfurt, Germany), with an adjusted significance level of alpha ≤ 0.05.

## 3. Results

### 3.1. Quantitative Results

The results obtained for the viable cell counts were tested if they were normally distributed using the Shapiro–Wilk test, which showed significant deviations from a normal distribution. Therefore, we decided to use the Kruskal–Wallis test for statistical evaluation, which revealed significant differences in the results. By performing Conover–Iman post hoc pairwise comparisons with Bonferroni–Holm corrections, we determined which compared pairs of groups showed significant differences while considering the risk of error.

**Figure 1:**
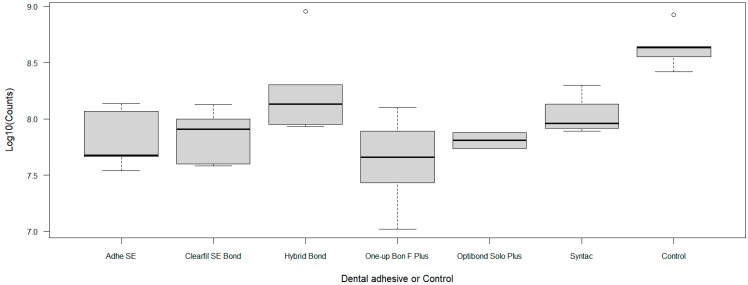
Cell counts of viable cells among the six groups treated with dental adhesives and the cell control group.

AdheSE (*p* = 0.0006), Clearfil SE Bond (*p* = 0.002), One-up Bond F Plus (*p* = 0.0001), and Optibond Solo Plus (*p* = 0.03) showed a statistically significant reduction in viable cells in comparison to the cell control after 48 h (Table 2). While Hybrid Bond offered a higher maximum cell count compared to the control group, the result was not statistically significant. No statistically significant differences could be observed between the different dentin adhesives due to partially high standard deviations (Figure 1).

Hybrid Bond applied sequentially, as recommended by the manufacturers, resulted in a statistically significant reduction in viable cells in comparison to a single application of Hybrid Brushes after 48 h (*p* = 0.01).

For One-up Bond F Plus, AdheSE, Clearfil SE Bond, and Syntac, no statistically significant differences between sequential and single application were observed after 48 h.

### 3.2. Qualitative Results

**Figure 2:**
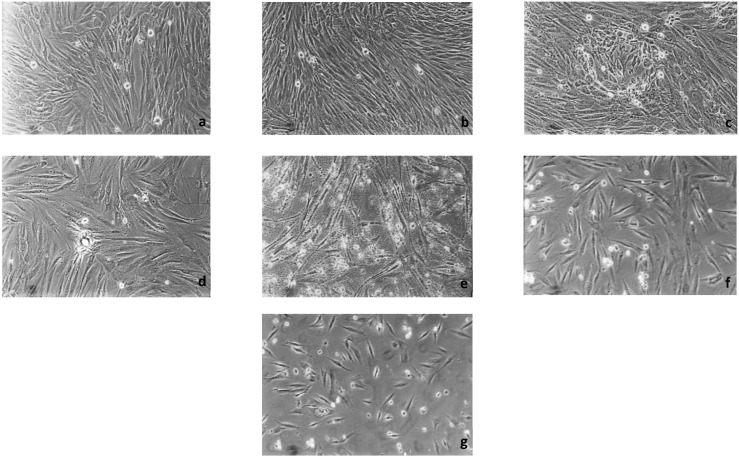
Cell control (**a**) and cell cultures with Hybrid Brushes (**b**), One-up Bond F Plus (**c**), AdheSE (**d**), Clearfil SE Bond (**e**), Syntac (**f**), and Optibond Solo Plus (**g**) after 48 h (100-fold magnification).

The results of qualitative evaluation are shown in Table 3, Table 4, Table 5, Table 6, Table 7, Table 8 and Table 9. Figure 2 depicts the characteristic appearance of the cell cultures under the influence of the different dentin adhesive materials. The morphological phenotype of this cell line is characterised by spindle-shaped, long cells derived from the gingiva (HGPFCs—human gingival primary fibroblast cells) representing human primary fibroblasts (pMF).

### 3.3. Reactivity Index Result

The reactivity index showed no statistically significant differences between the individual adhesives. A statistically significant difference was found for this parameter between each dentin adhesive and the cell control.

When applied sequentially as the manufacturers recommend, Hybrid Bond yielded a statistically significantly higher reactivity index relative to a single application of Hybrid Brushes after 48 h (*p* < 0.1 × 10^−6^).

When applied in single treatments, One-up Bond F Plus Agent A (*p* = 0.5 × 10^−3^) and Agent B (*p* = 0.03) yielded statistically significant increases in reactivity index values in comparison to sequential application of One-up Bond F Plus after 48 h.

We also found a statistically significantly higher reactivity index for AdheSE Bond when applied once relative to sequential application, as recommended by the manufacturers, after 48 h (*p* = 0.003). No statistically significant difference between Clearfil SE Bond applied sequentially and the single application of the adhesive was found over the observation period after 48 h. The reactivity index for sequentially applied Syntac was statistically significantly higher than that for a single application of Syntac Primer (*p* = 0.002).

According to our results, the null hypothesis H_0_^1^, i.e., that self-etch adhesives would show no differences relative to the etch-and-rinse adhesives, can be accepted.

Our null hypothesis H_0_^2^, i.e., that sequentially applied adhesive would have different cytotoxic effects relative to a single application of adhesive, can be accepted for Hybrid Bond, One-up Bond F Plus, AdheSE, and Syntac regarding the reactivity index and for Hybrid Bond regarding the quantitative evaluation.

## 4. Discussion

Materials that come into contact with living cells should be biocompatible; i.e., they should not cause cytotoxic, pro-inflammatory, mutagenic, or adverse immune reactions. Thus, cytotoxicity tests are crucial for evaluating the biocompatibility of materials. Other studies similar to ours researched time-dependent release of monomers due to incomplete polymerization [3,41]. This study design was adopted from our first study and is an extension from 24 to 48 h, thus enabling a direct comparison between the results [17].

The most important conclusion of the second part of our study is that there were no statistically significant differences regarding the cytotoxicity between the self-etch and etch-and-rinse dentin adhesives after 48 h. This finding is consistent with the first part of our study in terms of qualitative evaluation and reactivity index, and it is in accordance with findings from other studies [17,23,24,25,26,27,28]. Contradictorily, other studies have found that self-etch and etch-and-rinse dentin adhesives show greater cytotoxicity in comparison [16,23,29,32,34,35,36,44]. However, in all of the comparative studies performed by other researchers, different methods and materials were used. Notably, in the clinical application of etch-and-rinse adhesives, conditioning with 37% phosphoric acid is necessary. In contrast with our first investigation, this step has not been tested because there are already numerous studies addressing this topic [17,45,46].

First, we must highlight that the cytotoxicity of each dentin adhesive is linked to the cytotoxicity of the individual components of the substances, in which the concentrations of the different monomers play a significant role [47,48,49]. Adhesive systems usually contain a mixture of these monomers, which have already been proven to have cytotoxic and cell-modulating properties [16,50,51,52]. The typical components are HEMA, bis-GMA, UDMA, and TEGDMA, which have been shown to exhibit cytotoxic effects in a time- and concentration-dependent manner [41,53,54]. The widely accepted ranking of the cytotoxicity of these monomers, from highest to lowest, is as follows: Bis-GMA, UDMA, TEGDMA, and HEMA [47,51,55]. Bis-GMA can impair protein synthesis and induce reactive oxidative stress resulting in cell death, and it has been shown to be more toxic, even in small amounts, than other monomers [12]. HEMA, while accepted as being the least toxic, can delay cell cycle progression in fibroblasts by increasing the formation of reactive oxygen species (ROS) [56]. Furthermore, due to the hydrophilic character of small monomers like HEMA and TEGDMA, they can more easily penetrate through cell membranes [57,58,59]. The induced oxidative stress leads to mitochondrial dysfunction, which may lead to cellular damage, inflammatory responses, and caspase-mediated cell death (apoptosis) [12,60,61,62]. Apart from the monomers, free-radical-based photo-initiators are also expected to induce ROS formation [3]. Critically, the number of monomers in the eluates of adhesives do not exactly represent the real amount, since more hydrophilic monomers, such as TEGDMA, are more likely to be eluted than bis-GMA [63]. Nevertheless, all of our tested adhesives contain some or all of the above-mentioned monomers, which might explain the cytotoxic character observed in vitro according to the reactivity index. However, it must be stated that we did not study the specific intracellular pathways and therefore the potential linking of the cytotoxic effects of monomers to our results remains speculative. Further experiments are necessary in order to determine direct links.

As in the first part of the study, it must be stated that the solvents of the dentin adhesives also have an influence on their cytotoxicity [25,49]. The test materials are based on acetone, ethanol, and water. The water-based adhesives AdheSE, Clearfil SE Bond, and One-up Bond F Plus and the ethanol-based adhesive Optibond Solo Plus showed statistically significantly fewer viable cells compared to the cell control. For the acetone-based dentin adhesives, this effect could not be observed. As already explained, this might lead one to the conclusion that their cytotoxicity is hardly influenced by the individual composition and is instead influenced by the sum of their ingredients [25,40,49]. However, the effect of the previously mentioned water-based adhesives, which were all self-etch adhesives in our study, might depend on the fact that they contain acidic functional monomers, such as 10-Methacryloyloxydecyl dihydrogen phosphate (10-MDP; Hybrid Bond Base), 11-methacryloyloxyundecan-1,1-decarboxylic acid (MAC-10; One-up Bond F Plus Agent A), and 4-Methacryloxyethyl-trimellitic-anhydride (4-META; Clearfil SE Bond Primer). These monomers’ acidic nature might contribute to the adhesives’ cytotoxicity. It has been shown that 10-MDP promotes inflammatory responses and may suppress cell differentiation [64]. In contrast, 4-META has been shown to be more biocompatible, which might explain the less toxic effect of the acetone-based adhesive Hybrid Bond observed in our study [65]. Optibond Solo Plus is the only ethanol-based adhesive we tested. Thus, it is difficult to draw conclusions on its cytotoxic effect based on its solvent. It would be necessary to acquire more adhesives with the same solvent in order to determine whether there is a link to a potential cytotoxicity based on the solvent.

Along with AdheSE, Clearfil SE Bond and One-up Bond F Plus, Optibond Solo Plus showed a statistically significant reduction in viable cells compared to the cell control after 48 h. However, the cultures treated with Optibond Solo Plus did not show evidence of this effect after 24 h. One possible reason for this could be that the full extent of the cytotoxicity of Optibond Solo Plus only becomes apparent after 48 h; alternatively, it may be a product of the fact that cell changes take time before showing an effect. Similar observations were made in a study where the cytotoxicity of universal adhesives was tested after 24, 48, and 72 h. While cytotoxic effects were observed after 48 h and 72 h, no such effects arose after 24 h [20]. A possible explanation is that residual unpolymerized monomers are released over time.

When applied sequentially, Hybrid Bond resulted in statistically significantly fewer viable cells relative to a single application of Hybrid Brushes. Hybrid Brushes do not contain monomers, which might explain the low cytotoxicity. This effect was also mentioned in the first part of our investigation after 24 h [17]. Statistically significant differences were observed between the dentin adhesives AdheSE, Hybrid Bond, One-up Bond F Plus, and Syntac between parts treated sequentially and once, a finding that is in accordance with our first investigation [17]. In contrast, Clearfil SE Bond showed no statistically significant differences between sequential and single application over the observation term of 24 h. Cell regeneration that had already occurred could be a reason for this observation. Authors of recent studies made the same assumption after noting that the toxicity of adhesives appeared to decrease after 72 h [20].

The number of viable cells, a metric used in our multiparametric strategies, is not sufficient to draw conclusions about possible harmful cellular responses, as not all of them end in cell death [63].

Contrary to the quantitative assessment, every adhesive showed a statistically significant difference to the cell control regarding the qualitative evaluation, including with respect to the reactivity index, which was consistent after 24 h and 48 h [17]. Syntac, which is also acetone-based, presented one of the highest reactivity indices, even after 48 h, with no change compared to 24 h. This finding is also in accordance with other investigations, which classified Syntac as highly cytotoxic [33,36,40]. It is argued that Syntac contains a larger proportion of non-linked substances after polymerization; these substances can then be released to the culture medium over a prolonged period [40]. On the contrary, one study also found lower cytotoxicity of the dentin adhesive Syntac [66]. As mentioned in the first part of this study, the proportion of glutaraldehyde could play a role in the high cytotoxicity. Free glutaraldehyde is highly cytotoxic [33,44]. Most of the glutaraldehyde is bound irreversibly in the bonding process of the dentin adhesives. It stabilizes the collagen fiber network, which is exposed after etching. However, it has not yet been possible to determine how many molecules are free in the clinical application process [40].

Optibond Solo Plus remains the dentin adhesive with the highest reactivity index after 24 h and 48 h [17]. Regarding the components, Optibond Solo contains silica in different forms. They have been the subject of studies regarding possible cytotoxic effects. As studies have shown, silica might be responsible for cellular stress responses, which might cause cell death or promote transformation [67]. Those properties, combined with the cytotoxic effects of the other components of Optibond Solo Plus like bis-GMA, might be a possible explanation for the few viable cells and high reactivity index observed in this study [68].

It should be emphasized again that the experimental conditions used to apply the quantitative method can best be transferred to further investigations, thereby allowing the best possible comparison of different examinations. We consider this to be the most reliable method for evaluation, as qualitative assessments may vary from person to person. Therefore, it is important to integrate at least more than one observer to decrease subjectivity. In order to reduce bias, evaluations were performed according to predefined criteria and images were recorded for all samples. Formal interobserver agreement statistics were not performed but discrepancies between an experienced lab technician and a doctoral student were discussed and resolved by consensus. For future studies it is recommended to include interobserver agreement statistics, e.g., Cohen’s kappa.

In future research, the evaluation for these adhesives should be extended into a long-term trial conducted over 30 days, as described in 10993-5 [18]. In order to further reduce the cytotoxicity of the individual dentin adhesives, further investigation methods, such as MTT or WST-1 assays, are planned after screening. This investigation can also be complemented by further screening studies involving other adhesives to confirm the result of the comparison of the cytotoxicity of the self-etch and etch-and-rinse adhesives. Nevertheless, our study does not exactly mimic the environment in the oral cavity, constituting a major limitation. In vivo studies consider reactions of the surrounding tissue and cells, as well as the composition and temperature of human saliva, which is highly complicated to adapt in in vitro studies. However, cell culture models have gained value as an alternative to animal experiments, which are ethically controversial, costly, and time-consuming. These in vitro models are already widely accepted for use in restorative dentistry for testing the biocompatibility of materials, thereby further negating the need for animal experiments [69,70]. Undoubtedly, far more detailed studies regarding the mechanisms of toxicity, e.g., using immunohistological staining or flow cytometry, are necessary in order to foster a comprehensive understanding and allow the development of more biocompatible materials in the future [3,63]. Therefore, the possible mechanistic considerations that have been discussed regarding our experimental results, are mainly based on comparisons of previously published studies as our study did not directly assess possible mechanisms of cytotoxicity.

## 5. Conclusions

The present results show that the tested dentin adhesives were cytotoxic to the primary gingival fibroblasts in our multiparametric strategies. However, no differences between the cytotoxicity of the Self-Etch and Etch-and-Rinse adhesives could be found after 48 h. However, a phase in which the dentin adhesives exerted cytotoxic effects in our in vitro study was observed after 48 h. It should be emphasized that these are in vitro studies, and only an adequate measure of the hazard potential can be provided. There was also a difference in cytotoxic observations between the adhesives when applied sequentially and once. The trend in our results from our first screening study was confirmed by the longer observation period. To better understand and improve biocompatible material development, further research addressing mechanisms of toxicity is necessary.

## Figures and Tables

**Table 1:** Assessment of the reactivity index based on ISO 10993-5.

Grading	Reactivity	Condition of All Cultures
0	none	Discrete intracytoplasmatic granules were observed, and there was no cell lysis or reduction in cell growth.
1	slight	No more than 20% of the cells were round and loosely attached without intracytoplasmatic granules or showed changes in morphology; lysed cells were sporadically present; and only slight growth inhibition was observed.
2	mild	No more than 50% of the cells were round or devoid of intracytoplasmatic granules or exhibited extensive cell lysis; no more than 50% growth inhibition was observed.
3	moderate	No more than 70% of the cell layers contained rounded cells or had been lysed; cell layers were not completely destroyed, but more than 50% growth inhibition was observed.
4	severe	Nearly complete or complete destruction of the cell layers was observed.

**Table 2:** Cell counts of viable cells, showing minimum (Min.), maximum (Max.), and median values of the six dentin adhesives after 48 h. *n*= number of tests.

No.	Dental Adhesive (*n* = 8)	Min.	Max.	Median	Significance in Rel. to No.
1	AdheSE	34,640,000.00	136,900,000.00	47,375,000.00	7
2	Clearfil SE Bond	38,280,000.00	134,200,000.00	82,885,000.00	7
3	Hybrid Bond	86,440,000.00	906,100,000.00	136,100,000.00	-
4	One-up Bon F Plus	10,480,000.00	126,800,000.00	46,205,000.00	7
5	Optibond Solo Plus	54,740,000.00	75,870,000.00	65,305,000.00	7
6	Syntac	77,690,000.00	200,200,000.00	91,895,000.00	-
7	Cell Control	263,300,000.00	846,300,000.00	430,600,000.00	1, 2, 4, 5

**Table 3:** Qualitative evaluation of Hybrid Bond.

Concentration	Components Treated with Sequentially Applied Adhesive	Components Treated with a Single Application of Adhesive	
		Hybrid Base	Hybrid Brushes
I	0.1–2.0 µL: few viable fibroblasts, no mitosis, material widely distributed across the bottom of the Petri dish, up to 100% cell death	0.04–2.0 µL: rounded fibroblasts, mitosis, material widely distributed across the bottom of the Petri dish, less dense fibroblast lawn, up to 100% cell death	0.1–1.0 µL: like the cell control
II	2.5–5.0 µL: material spread very widely across the bottom of the Petri dish, 100% cell death	3.0–5.0 µL: material spread very widely across the bottom of the Petri dish, 100% cell death	1.1–3.4 µL: like concentration I

**Table 4:** Qualitative evaluation of One up Bond F Plus.

Concentration	Components Treated with Sequentially Applied Adhesive	Components Treated with a Single Application of Adhesive	
		Agent A	Agent B
I	1.0–6.0 µL: few rounded fibroblasts, little mitosis, material widely distributed on the bottom of the Petri dish, dense fibroblast lawn	1.0–4.0 µL: rounded fibroblasts, up to 100% cell death	1.0–5.0 µL: rounded fibroblasts, cells that appear to be partially still viable, fibroblasts growing on material, fibroblast lawn that is not so dense
II	7.0–14.0 µL: rounded fibroblasts, fibroblast lawn that is less dense than the cell control	5.0–8.0 µL: rounded fibroblasts, less mitosis, few viable cells, up to 100% cell death	6.0–10.0 µL: few fibroblasts that appear viable, fibroblasts growing on material, material mainly distributed on the bottom of the Petri dish

**Table 5:** Qualitative evaluation of AdheSE.

Concentration	Components Treated with Sequentially Applied Adhesive	Components Treated with a Single Application of Adhesive	
		AdheSE Primer	AdheSE Bond
I	2.5–6.0 µL: hardly any mitosis, viable fibroblasts, slightly less dense fibroblast lawn relative to the cell control	5.0–9.0 µL: viable fibroblasts, mitosis, slightly less dense fibroblast lawn relative to the cell control	3.0–4.0 µL: many rounded cells, viable fibroblasts at the border of the Petri dish, fibroblast lawn that is not so dense
II	7.0–12.0 µL: like concentration I	10.0–14.0 µL: like concentration I	5.0–7.0 µL: 100% cell death

**Table 6:** Qualitative evaluation of Clearfil SE Bond.

Concentration	Components Treated with Sequentially Applied Adhesive	Components Treated with a Single Application of Adhesive	
	Clearfil SE Bond	Clearfil SE Bond Primer	Clearfil SE Bond
I	2.0–3.0 µL: many dead fibroblasts, some seemingly viable atypical cells, 75–95% cell death	4.0–5.0 µL: few viable fibroblasts, material spread widely across the bottom of the Petri dish, up to 100% cell death	3.0–4.0 µL: viable and many dead cells, fibroblast lawn that is less dense than the cell control
II	4.0–5.0 µL: 90–100% cell death	6.0–8.0 µL: 100% cell death	5.0–6.0 µL: 100% cell death

**Table 7:** Qualitative evaluation of Syntac.

Concentration	Components Treated with Sequentially Applied Adhesive	Components Treated with a Single Application of Adhesive		
		Syntac Primer	Syntac Adhesive	Syntac Heliobond
I	0.1–1.0 µL: few viable fibroblasts, rounded cells, material spread widely across the bottom of the Petri dish, 99–100% cell death	1.0–6.0 µL: many viable cells, dead fibroblasts, fibroblast lawn that is not so dense	0.2–2.0 µL: few viable cells, rounded fibroblasts, mitoses, fibroblast lawn that is not very dense	1.0–5.0 µL: rounded and viable cells, many dead cells, fibroblast lawn that is not very dense
II	2.0–2.5 µL: some small viable fibroblasts, most cells dead, material spread very widely across the bottom of the Petri dish, 90–100% cell death	7.0–12.0 µL: many rounded cells, less dense fibroblast lawn than the cell control	3.0–5.0 µL: 100% cell death	6.0–10.0 µL: many rounded cells, up to 100% cell death

**Table 8:** Qualitative evaluation of Optibond Solo Plus.

Concentration	Optibond Solo Plus
I	1.0–4.0 µL: many viable cells, rounded fibroblasts, vacuolated cells and material spread widely across the bottom of the Petri dish, first fibroblasts grow on material, less dense fibroblast lawn than the cell control
II	5.0–8.0 µL: material spread widely across the bottom of the Petri dish, 100% cell death

**Table 9:** Reactivity index values of dental adhesive groups and control groups. Mean, standard deviation (sd), minimum (Min.), maximum (Max.), and median values of the six dentin adhesives after 48 h are shown (corresponding to the reactivity index).

No.	Dental Adhesive (1–6)	Mean	sd	Min.	Max.	Median	Significance in Rel. To No.
1	AdheSE	2.94	1.03	1.00	4.00	3.00	7
2	Clearfil SE Bond	3.22	0.96	1.00	4.00	4.00	7
3	Hybrid Bond	2.66	1.63	0.00	4.00	4.00	7
4	One-up Bond F Plus	2.94	1.03	1.00	4.00	3.00	7
5	Optibond Solo Plus	3.53	0.84	2.00	4.00	4.00	7
6	Syntac	3.16	0.76	2.00	4.00	3.00	7
7	Cell control	0.00	0.00	0.00	0.00	0.00	1–6

## Data Availability

The original contributions presented in this study are included in the article. Further inquiries can be directed to the corresponding authors by mail.
